# Digital Twins and Offline Reinforcement Learning for Mechanical Circulatory Support Decisions: What the Current Evidence Can and Cannot Support

**DOI:** 10.7759/cureus.117234

**Published:** 2026-09-30

**Authors:** Oscar Mingtian Li, Yijiang Li, Summer Xia Tian Li

**Affiliations:** 1 Zweistein Innovation Lab, Active Care and Education in Sports and Outreach (ACESO) Foundation, Dublin, USA

**Keywords:** cardiogenic shock, clinical decision support, digital twin, extracorporeal membrane oxygenation, heart failure, mechanical circulatory support, myocardial recovery, offline reinforcement learning, off-policy evaluation, ventricular assist device

## Abstract

Mechanical circulatory support (MCS) is not a single intervention but a sequence of decisions: whether to initiate support, which device to use, how to titrate it while other therapies change, when to test for recovery, and whether to wean, escalate, bridge, or withdraw. Each decision has its own eligible population, time origin, action set, and cost of error. Interest is growing in patient-specific digital twins and offline reinforcement learning to support these decisions, and the first device-specific probabilistic twins and safety-regularized weaning policies have now been published. This narrative review examines what that literature currently establishes. Cardiac digital twins have produced their most convincing results in electrophysiology and resynchronization, where the modeled substrate is anatomically stable, and the intervention is discrete; circulatory support is harder because it involves a continuously titrated device acting on a rapidly changing patient under unrecorded co-interventions. The published MCS work is small, largely single-device, restricted to patients already receiving support, and evaluated against hemodynamic surrogate rewards inside simulators estimated from the same trajectories used for training. Three problems recur and appear to be the limiting factors rather than model capacity: predictive accuracy for an endpoint does not establish that an action is beneficial; a simulator cannot independently certify a policy optimized against it; and information available to a retrospective analyst is not information that was available at the decision time. The defensible near-term claim is improved state estimation and forecasting with calibrated uncertainty and explicit abstention. The initiation question requires target-trial emulation rather than a post-implant cohort, and public critical-care datasets can validate chronology and transport but cannot validate device titration or weaning policy.

## Introduction and background

Mechanical circulatory support (MCS) is usually described through its endpoints: survival, myocardial recovery, transplantation, explantation, or successful weaning. The decisions that produce those endpoints are trajectories rather than single choices. A team recognizes a syndrome and grades its severity, decides whether support is indicated at all, selects and initiates a device, adjusts the level of support while inotropes, vasopressors, ventilation, volume, and anticoagulation all change around it, tests whether the native heart is recovering, and then weans, escalates, bridges to a durable device or transplant, or moves to comfort-focused care. Contemporary guidelines and scientific statements treat these as related but distinct decisions, with different eligibility, different evidence, and different consequences of error [1-7].

This structure matters for any computational system intended to help. A model that accurately predicts a favorable terminal outcome can still be clinically unhelpful or harmful if it uses information that was not available when the decision had to be made, if it responds after the window in which action would have mattered, or if it recommends an action that was rarely or never taken in comparable patients and therefore has no observational support. Discrimination for an endpoint and usefulness at a decision node are different properties, and one does not imply the other.

Two computational approaches have attracted particular attention. The first is the patient-specific digital twin: a model that maintains a latent physiological state, updates it as new observations arrive, forecasts how that state will evolve under candidate actions, and reports uncertainty [8,9]. The second is offline reinforcement learning, which attempts to learn a treatment policy from historical trajectories without experimenting on patients [10-12]. The two have recently converged on circulatory support: probabilistic twins of pump-level physiology have been proposed for smart weaning, and safety-regularized offline policies have been trained and evaluated against those twins [13-15].

That convergence is a reasonable place to ask what has actually been established. The purpose of this review is not to catalog architectures. It is to separate what the current literature supports from what it is often read as supporting, and to state which evidence would be needed to move each claim up one level. We take the position that the binding constraints on this field at present are not model capacity or data volume but three specific evidentiary problems: the gap between prediction and action benefit, the circularity of certifying a policy against a simulator built from the same data, and the difference between information available retrospectively and information available at the decision time.

## Review

Review methodology and scope

This is a narrative review, not a systematic review, and it is reported with reference to the Scale for the Assessment of Narrative Review Articles (SANRA) criteria for narrative review quality rather than the Preferred Reporting Items for Systematic Reviews and Meta-Analyses (PRISMA) guidelines [16]. No protocol was registered, and no quantitative synthesis was performed because the included literature spans clinical trials, prediction-model studies, machine-learning methods papers, and reporting guidance, which share no common effect measure.

The reference base was assembled in two stages. An initial corpus was compiled by the authors during earlier work on this topic. It was then supplemented by targeted searching of PubMed-indexed literature and by general web search, both performed on September 8 and 9, 2026, with citation chaining from the retrieved articles. Searches were run by a single reviewer without blinding. Because a substantial part of the digital twin and offline reinforcement learning literature appears as conference proceedings, workshop papers, and preprints that are not indexed in PubMed, general web search rather than PubMed was the route by which that part of the corpus was identified.

Searches were executed as free-text Boolean queries run iteratively rather than as a single locked strategy. The queries were combinations of three term blocks. Block A, support modality: "mechanical circulatory support" OR "ventricular assist device" OR "extracorporeal membrane oxygenation" OR "Impella" OR "micro-axial flow pump" OR "cardiogenic shock." Block B, computational approach: "digital twin" OR "reinforcement learning" OR "offline reinforcement learning" OR "off-policy evaluation" OR "machine learning" OR "prediction model." Block C, decision node: "weaning" OR "explantation" OR "myocardial recovery" OR "ramp test" OR "titration" OR "candidacy" OR "initiation." The principal queries were (A) AND (B), (A) AND (C), and (A) AND (B) AND (C). Trials, reporting guidance, and public critical-care datasets named within the retrieved articles were then retrieved individually by name.

Selection was purposive rather than protocol-driven. Articles were included when they defined a decision node, reported a clinical trial or registry result bearing on one, introduced a modeling method used in this domain, or set out reporting or regulatory expectations. Non-English articles, conference abstracts without an accessible full record, and articles addressing respiratory support only were not sought. Screening was performed in a single pass by one reviewer: titles and abstracts of retrieved records were read, candidates were retrieved in full text or, for conference papers, as the full proceedings record, and inclusion was decided against those criteria. There was no second screener, no adjudication procedure, and no risk-of-bias instrument.

Because the search was iterative and purposive rather than executed once against a locked protocol, no screening log was kept, and the numbers of records identified and screened at each stage were not recorded prospectively. They are therefore not reported here, since any figure reconstructed after the fact would not describe what actually occurred. What can be stated exactly is the endpoint: 73 references are cited, of which three are the support-specific digital twin, simulator, or learned-policy studies whose design characteristics are set out in Table 1. Two further computational studies on adjacent questions are discussed in the text but are not included in Table 1: a registry prediction model of myocardial recovery on durable support, which estimates risk without simulating physiology or learning a policy, and an offline reinforcement learning study in venovenous extracorporeal support for respiratory failure, which concerns respiratory rather than circulatory support.

**Table 1:** Design characteristics and principal limitations of the support-specific digital twin, simulator, and learned-policy studies in mechanical circulatory support. Studies are those cited in this review that develop or evaluate a digital twin, simulator, or learned treatment policy using data from patients on mechanical circulatory support. A registry prediction model of myocardial recovery and an offline reinforcement learning study in venovenous extracorporeal support for respiratory failure are discussed in the text but are not included because the first is a prediction model and the second concerns respiratory rather than circulatory support. Entries record what each study reports; fields not reported are marked as such rather than inferred. Sources: [13-15].

Study	Population and device	Sample size	Decision or quantity modeled	Outcome or reward	Validation approach as reported	Principal limitation for decision-support claims
Sun et al., 2024 [14]	Patients undergoing high-risk percutaneous coronary intervention supported with a percutaneous micro-axial flow pump	211 patients; 43,814 thirty-minute time series	Forward simulator of the aortic pressure response to pump support level; no policy is learned	Predicted mean aortic pressure with calibrated uncertainty	Each training batch split into context and target points; no patient-level held-out cohort and no external dataset reported	This is a forecasting model only. The authors state that further clinical metrics are required before generalizability can be assessed.
Sun et al., 2025 [13]	Cardiogenic shock patients supported with percutaneous micro-axial flow pumps	Not reported in the workshop paper	Probabilistic Transformer digital twin of circulatory dynamics, then used as the environment in which offline reinforcement learning methods are benchmarked.	Forecast error against baseline dynamics models; offline reinforcement learning benchmarked on clinically framed metrics	Twin compared against baseline models; candidate policies evaluated inside the twin.	Policy comparison occurs entirely within a learned simulator. Because this is a workshop paper, the depth of peer review is limited.
Tumay et al., 2026 [15]	Cardiogenic shock patients supported with percutaneous micro-axial flow pumps	379 patients, mean record 65.5 hours, 12 device-derived features	Weaning: choice of discrete pump support level	Composite of a hemodynamic reward, a penalty on abrupt action changes, and a weaning score conditioned on hemodynamic stability	Patients split 65:15:20 into training, validation, and testing sets for the dynamics model; policy value then estimated by model-based off-policy evaluation inside that learned twin, with additional synthetic trajectories rolled out from it.	Reward components are author-designed proxies that the authors state await clinician review. The policy is never valued outside a model fitted to its own training distribution.

Every reference in this article was individually checked against a retrieved bibliographic record for author, title, journal, year, volume, pages, and digital object identifier before inclusion. Five candidate references that could not be confirmed, or whose retrieved record contradicted the citation, were corrected or removed during this process. Readers should note the limitations that follow from a purposive, single-reviewer search: the corpus is not exhaustive, and the risk of selection favoring work consistent with the authors' argument cannot be excluded.

MCS is a sequence of distinct decisions

It is useful to name the decision nodes explicitly, because much of the confusion in this literature comes from treating them as one problem. Candidacy or initiation asks whether a patient who is not yet supported should receive a device and, if so, which one. Its eligible population includes every support-eligible patient, including those who were never implanted. On-support management asks how to titrate flow or speed, how to coordinate that with inotropes, vasoactive drugs, ventilation, volume, and anticoagulation, and when to escalate, combine devices, or recognize futility. Its population is patients already receiving support. Recovery assessment asks whether the native ventricle has improved enough to be tested, typically through structured ramp or low-support protocols [17,18]. Weaning or explantation asks whether to continue support, step down, remove the device, bridge to a durable device or transplant, or transition to palliation [19,20].

These nodes have genuinely different evidence bases. Structured Impella weaning protocols and echocardiographic ramp testing are described in single-center series and algorithmic proposals rather than randomized trials [18,19,21]. Myocardial recovery on durable support has a longer literature, from the early Harefield combination-therapy work through RESTAGE-HF, with registry analyses showing that sustained recovery sufficient for explantation is uncommon and that the opportunity may be under-recognized [22-28]. Durable device outcomes themselves have been transformed by fully magnetically levitated pumps [29]. A model trained and validated at one node does not transfer to another simply because both involve the same device. Table 2 sets out, for each node, the population in which the question arises, the strongest evidence currently available, and what remains unestablished.

**Table 2:** Decision nodes in mechanical circulatory support, the evidence available at each node, and what remains unestablished. The four decision nodes are those defined in the text. Evidence cited in the third column is the strongest identified in this review and is not exhaustive. Sources: [13-15,17-35]. Digital twin, simulator, and learned-policy studies cited in the third column are described in full in Table 1. ECLS, extracorporeal life support; ECMO, extracorporeal membrane oxygenation; IABP, intra-aortic balloon pump; DanGer Shock, Danish-German Cardiogenic Shock trial; RESTAGE-HF, Remission from Stage D Heart Failure

Decision node	Population in which the question arises	Strongest evidence currently available	Not established
Candidacy and initiation	All support-eligible patients, including those never implanted	Randomized trials of initiation strategies in infarct-related cardiogenic shock: IABP-SHOCK II was neutral, including at six years; ECLS-SHOCK and ECMO-CS were neutral; DanGer Shock reduced 180-day mortality, with a higher rate of serious adverse events; an individual patient data meta-analysis of temporary support [30-35]	Which individual patient benefits. No support-specific computational study addresses this node because every cohort in Table 1 is conditioned on the implantation decision.
On-support management: titration and co-intervention	Patients already receiving support	Probabilistic digital twins and offline reinforcement learning applied to micro-axial flow-pump support levels, with policies evaluated inside learned simulators [13-15]	Whether any titration policy improves patient outcomes. The rewards optimized are hemodynamic surrogates, not survival, recovery, or complication burden.
Recovery assessment	Patients already receiving support	Structured ramp and low-support protocols described in single-center series and algorithmic proposals rather than randomized trials [17,18,21]	Prospective or external validation of any recovery-prediction model, with calibration reported alongside discrimination.
Weaning, explantation, and bridging	Patients already receiving support	Weaning and explantation literature from the early Harefield combination-therapy work through RESTAGE-HF, with registry analyses showing sustained recovery to be uncommon and possibly under-recognized [19,20,22-28]; durable device outcomes transformed by fully magnetically levitated pumps [29]	Optimal timing for an individual patient. No learned weaning policy has been run prospectively, silently or otherwise.

What the randomized evidence does and does not settle

Any claim that an algorithm can improve MCS decisions has to be positioned against what randomized trials have shown, which is more specific and more sobering than it is often rendered. In infarct-related cardiogenic shock, intra-aortic balloon counterpulsation did not reduce mortality in IABP-SHOCK II, and the neutral result persisted at six years [30,31]. Routine early venoarterial extracorporeal life support did not reduce 30-day mortality in ECLS-SHOCK, and immediate extracorporeal membrane oxygenation did not improve the composite outcome in ECMO-CS [32,33]. A microaxial flow pump did reduce 180-day all-cause mortality in DanGer Shock, at the cost of a significantly higher rate of serious adverse events, including bleeding, limb ischemia, and renal replacement therapy [34]. An individual patient data meta-analysis of randomized trials of temporary support in this population has since been reported [35].

Three points follow. First, these are average treatment effects in defined populations under defined strategies; none of them licenses a general statement that a device class is good or bad. Second, the DanGer Shock result in particular is a benefit-and-harm result rather than a clean win, which is precisely the situation in which per-patient decision support is attractive and also the situation in which a miscalibrated recommendation does the most damage. Third, and most important for this review, none of these trials answers the question that a titration or weaning algorithm is trying to answer. They compare strategies at initiation; they do not establish what the optimal support level is for a given patient 18 hours into support, nor when that patient should be tested for recovery.

What a digital twin must specify to earn the name

The term digital twin is applied loosely enough that it is worth stating what the concept requires. A latent vector attached to a patient record is not a twin. A usable formulation specifies a latent physiological state, an observation model linking that state to what is measured, a transition model describing how the state evolves, an explicit action set that can alter transitions, a mechanism for updating patient-specific parameters as data arrive, and calibrated uncertainty in its forecasts [8,9].

For circulatory support, the candidate state variables are reasonably well agreed upon: contractility, preload, afterload, systemic and pulmonary vascular resistance, ventricular interdependence, congestion, end-organ perfusion, and some representation of a recovery regime. Observations include device telemetry, invasive pressures and flows, laboratory values, echocardiography, electrocardiography, and clinical documentation. The action set must include support-level changes and, critically, the co-interventions that are changed at the same time. The quantity of interest is the posterior distribution over physiological state conditional on the information available at a decision node, which is not the same object as a retrospective summary of the whole admission. That distinction sounds pedantic and is not: it is the difference between a model that could have been run at the bedside and one that could not.

Where cardiac digital twins have actually delivered

The strongest cardiac digital twin results to date come from electrophysiology and resynchronization. Personalized virtual-heart models built from imaging have been used to identify ablation targets in infarct-related ventricular tachycardia [36], and patient-specific models have been used to predict left ventricular reverse remodeling after cardiac resynchronization therapy by virtually pacing the model [37]. These are genuine successes, and it is worth being precise about why they were tractable. The modeled substrate is anatomically stable over the relevant horizon, the intervention is discrete and well specified, the model is personalized from a rich imaging study, and the prediction target is a remodeling outcome measured months later rather than a minute-to-minute control decision.

Circulatory support inverts most of these favorable conditions. The substrate changes hour by hour, the intervention is continuously titrated rather than discrete, personalization must come from streaming telemetry rather than a single imaging study, and the decisions of interest occur on a scale of hours. A recent scoping review of digital twin applications in adult critical care found the field still largely at the level of development and feasibility work rather than implementation [38]. Enthusiasm for the electrophysiology results should therefore be discounted when transferred to the circulatory support setting.

Sequence models and the limits of endpoint prediction

A large methodological literature exists for irregular longitudinal clinical data, and it provides the baselines against which any twin should be judged. Recurrent architectures handling missingness, attention-based interpretable models, transformer encoders for electronic health records, and multi-horizon forecasting architectures all address parts of the problem [39-42]. Dynamic survival models produce updated risk estimates as new observations arrive [43,44], and joint longitudinal and time-to-event models remain a strong, interpretable statistical reference point [45]. Classical state-space and hidden-state approaches are the simplest credible starting points and are too often skipped [46,47]. Self-exciting point processes and distributed-lag models offer principled ways to handle event clustering and delayed effects in irregularly observed data [48,49]. Within MCS specifically, machine learning has been applied to predict myocardial recovery resulting in pump explantation among 20,270 patients with durable continuous-flow left ventricular assist devices in the Interagency Registry for Mechanically Assisted Circulatory Support (INTERMACS), with an area under the receiver operating characteristic curve of 0.813 to 0.824 on an internal 70:30 split, exceeding established recovery risk scores at 0.744 to 0.796 [50]. That study is a prediction model rather than a digital twin, simulator, or learned policy: it estimates the probability of an outcome, does not model how physiology responds to an action, and reports discrimination without calibration or external validation.

The recurring evaluation failure in this area is the use of area under the receiver operating characteristic curve as the primary or sole metric. For a probabilistic forecasting system, this is inadequate: it is insensitive to calibration, it says nothing about interval coverage, it is computed over a whole admission rather than at a decision node, and it can be high while the model is systematically late. Proper scoring rules, which are minimized only by the true predictive distribution, are the appropriate primary criterion, supplemented by calibration curves, interval coverage, and multi-step forecast error at clinically meaningful horizons [51]. A twin earns an engineering claim only when it beats strong mechanistic, state-space, and sequence baselines on those measures, on held-out patients, and preferably on external data.

Offline reinforcement learning for device weaning, and what it currently shows

Reinforcement learning has been applied to sequential treatment problems in critical care for several years, most visibly in sepsis, and the methodological cautions were articulated early [10-12]. Recent work has extended the approach to venovenous extracorporeal membrane oxygenation for respiratory failure, jointly optimizing ventilator and extracorporeal settings in 184 adults at a single tertiary center [52]. That study concerns respiratory rather than circulatory support and is cited here as the nearest methodological analogue, not as evidence about circulatory support decisions. The MCS-specific line of work is newer and small: a data-driven simulator for MCS built with domain-adversarial neural processes [14], a probabilistic digital twin proposed for data-driven smart weaning [13], and a guardian-regularized safe offline reinforcement learning method for weaning that constrains the learned policy toward the region supported by observed data [15].

The design characteristics of this support-specific literature, including the cohorts, rewards, and validation approaches on which it rests, are set out in Table 1. This work is careful and is honestly reported by its authors, and the criticisms that follow are about what the evidence can bear, not about the quality of the modeling. Four constraints apply. The cohorts are small and drawn from patients already receiving support from a single device family, so nothing in them speaks to who should have been implanted. The reward functions are hemodynamic surrogates rather than patient outcomes, so a reported improvement in reward is an improvement in a proxy whose relationship to survival, recovery, or complication burden is assumed rather than demonstrated. The policies are evaluated in simulators estimated from the same trajectories that trained them, which is discussed below. And there has been no prospective deployment, silent or otherwise, and therefore no evidence about clinician interaction, alert burden, or behavior change.

Why a simulator cannot certify the policy trained against it

The most consequential methodological issue in this literature is circularity. If a transition model is estimated from a set of observed trajectories, and a policy is then optimized to perform well under that transition model, evaluating the policy in the same model measures the consistency of the pipeline with itself. It does not provide independent evidence of clinical benefit. Wherever the learned policy departs from historical practice, which is exactly where its value would lie, the simulator is extrapolating into state-action regions it never observed, and its error there is unconstrained.

It is therefore material whether the cited support-specific studies evaluated their policies against data independent of the data used to build their simulators, and on the published record, none of them did in the sense that matters. In the micro-axial flow pump work, patients were split 65:15:20 into training, validation, and testing sets, so the transition model was fitted on one set of patients and its forecasting accuracy assessed on held-out patients, which is a genuine held-out evaluation of the twin [15]. Policy value, however, was then estimated by model-based off-policy evaluation inside that same learned twin, supplemented by trajectories rolled out from it, rather than against independent real trajectories or an independent value estimator [13,15]. The earlier simulator paper reports no patient-level held-out cohort and no external dataset, splitting each training batch into context and target points instead [14]. The respiratory analogue shows the same pattern: in the venovenous extracorporeal work, fitted Q evaluation was used, and the learned policy was compared with clinician action distributions on the same single-center cohort from which it was learned [52]. Held-out patients therefore support the claim that these dynamics models forecast well; they do not support the claim that the learned policies would act well, because no policy in this literature has yet been valued outside a model estimated from its own training distribution.

Model-based offline policy optimization methods address this explicitly, typically by penalizing the objective in proportion to model uncertainty so that the policy is discouraged from exploiting regions where the dynamics model is unreliable [53]. Off-policy evaluation estimators, including doubly robust constructions that combine importance weighting with a learned value model, give partially independent value estimates but degrade as the learned policy diverges from the behavior policy, and their variance in long-horizon clinical problems is often severe enough to make the estimate uninformative [54]. The practical implications are that behavior-policy overlap must be reported rather than assumed, that value estimates should be accompanied by the effective sample size on which they rest, and that a policy that recommends an action never observed in similar patients should abstain rather than recommend.

Information availability, leakage, and the problem of three clocks

A separate failure mode is temporal rather than causal. Retrospective clinical records collapse several distinct times into one. The time an event physiologically occurred, the time its result first became usable by a clinician or system, and the time it was entered into the record are different, and in intensive care they can differ by hours. A laboratory value timestamped at the moment the specimen was drawn is not knowable at that moment; a note documenting a deterioration is written after the deterioration. Any model trained on a naive extraction that uses recorded time as though it were available time has been given information from the future, and its apparent performance is inflated by an amount that is generally not estimable after the fact.

This is not a hypothetical concern in a domain where the whole proposition is timeliness. A weaning algorithm that appears to anticipate decompensation may simply be reading documentation created because the decompensation had already been recognized. Guarding against it requires that each event carry event time, available time, and recorded time separately; that features at a decision node be constructed only from what was available at that node; and that validation split patients, sites, and calendar periods rather than randomly splitting observations. Negative-control outcomes and exposures, and deliberate future-information oracles that quantify how much performance a leak would buy, are practical diagnostics [55].

The initiation question requires a target trial, not a post-implant cohort

The most common category error in this field is using a cohort of supported patients to make claims about whether support should be given. A post-implant cohort is conditioned on the implantation decision. It contains no patients who were eligible and not implanted, and therefore contains no information about the counterfactual. No amount of modeling recovers a comparison group that was never sampled.

The appropriate framework is target-trial emulation: specify the eligibility criteria, the time zero, the treatment strategies being compared, the assignment procedure, the outcome, and the follow-up, and then emulate that protocol in observational data, with time-varying confounding handled by methods designed for it, such as marginal structural models [56,57]. Doing this for MCS initiation requires a cohort assembled at the point of eligibility rather than at the point of implantation, and it requires that the co-interventions and clinical reasoning that drove the implantation decision be recorded well enough to be adjusted for. Psychosocial evaluation is a formal part of candidacy assessment for durable support and is associated with outcomes, yet is rarely available in structured form in the datasets used for modeling, which is a concrete example of a determinant of the treatment decision that is systematically missing from the data used to study it [58,59].

What public critical-care datasets can and cannot validate

Public intensive-care datasets have been indispensable to methodological progress. MIMIC-IV, the eICU Collaborative Research Database, and HiRID provide large, well-documented, differently structured cohorts that support development, multicenter held-out evaluation, and higher-frequency physiological work, respectively [60-63]. Emerging common data standards and new cardiovascular longitudinal cohorts make cross-source work more tractable [64,65].

Their limits for this specific problem should be stated plainly. They do not contain synchronized device telemetry at the resolution at which support is actually titrated; they do not record the alternative actions that were considered and rejected; they capture co-interventions incompletely, and they contain no expert-defined set of acceptable actions against which a policy could be judged. They can therefore support claims about state estimation, event recognition, chronology handling, and transport across sites. They cannot validate device-specific initiation, titration, or weaning policy, and a paper that trains a weaning policy on them is making a claim its data cannot carry. Governed multicenter telemetry with synchronized co-interventions and adjudicated outcomes is the missing resource, and its absence, rather than any algorithmic limitation, is what currently caps the field.

Reporting standards, evaluation design, and the regulatory frame

The reporting infrastructure for this work already exists and is underused. TRIPOD+AI covers prediction models developed with regression or machine learning [66], PROBAST+AI provides the corresponding risk-of-bias and applicability assessment [67], DECIDE-AI covers early-stage live clinical evaluation of decision support systems [68], and SPIRIT-AI and CONSORT-AI cover protocols and reports of trials of AI interventions [69,70]. An American Heart Association scientific statement sets out expectations for artificial intelligence in cardiovascular care [71], and general guidance on benchmarking medical AI agents has recently been published [72]. For any system intended to inform device management, the United States Food and Drug Administration's guidance on assessing the credibility of computational modeling and simulation in device submissions defines the question a regulator will ask: what is the specific context of use, and what evidence establishes credibility for that context [73].

Read together, these documents describe an evidence ladder that the current MCS literature has climbed only the first few rungs of. State estimation and forecasting can be established retrospectively with proper scoring rules and external validation. Policy value cannot, and requires overlap-aware off-policy evaluation with honest uncertainty, followed by silent prospective running in which the system's recommendations are recorded but not shown. Only then do human-factors and workflow studies become meaningful, and only after those does a randomized or stepped-wedge evaluation against patient outcomes become appropriate. Skipping rungs is the failure mode to watch for, and the clearest warning sign in a manuscript is a policy-value claim supported only by simulator performance.

Separating what circulatory support evidence shows from what methodology recommends

Because this review draws on two distinct bodies of work, it is worth marking which conclusions rest on which. Four of its claims are supported directly by MCS evidence. First, the randomized trials establish average effects of initiation strategies in defined populations and establish nothing about titration or weaning, which follows from the trial protocols themselves [30-35]. Second, the support-specific computational literature is confined to cohorts of patients already receiving support, which follows from the cohorts as reported (Table 1) and is what makes initiation claims unavailable to it. Third, the reward functions used in that literature are hemodynamic surrogates rather than patient outcomes, which is stated by the authors of those studies [13,15]. Fourth, no system in this literature has been run prospectively, silently or otherwise, which follows from the absence of any such report.

The remaining positions taken here are methodological recommendations imported from the offline reinforcement learning, causal inference, and prediction-model literatures, and they are not findings about MCS. That a policy should abstain outside the support of the behavior policy, that off-policy value estimates should be reported with the effective sample size on which they rest, that proper scoring rules should displace area under the receiver operating characteristic curve as the primary forecasting criterion, that event time and available time must be recorded separately, and that initiation questions require target-trial emulation are general results applied here by analogy [51,53-57]. They are, in the authors' view, the right requirements to impose on this field, but they have not themselves been tested in MCS. A reader is entitled to accept the first set of claims while disputing the second.

Limitations

Several limitations should temper the conclusions drawn here. The limitations of the search and selection process are set out in the methodology section above and are not repeated here. The MCS-specific digital twin and offline reinforcement learning literature is small and rapidly moving, and part of it exists as conference proceedings and workshop papers rather than journal articles, which limits both the depth of peer review behind it and the stability of the record. The review is restricted to circulatory support and does not systematically address respiratory extracorporeal support, which is cited only where it is the nearest available analogue [52]. Finally, the argument advanced here is methodological; it identifies what current evidence supports but does not itself provide new empirical evidence, and the evaluation program it implies has not been executed.

## Conclusions

In its published record, the support-specific digital twin and offline reinforcement learning literature for MCS establishes improved patient-specific state estimation and short-horizon forecasting with calibrated uncertainty, but nothing yet about whether a learned policy benefits patients. The requirements for a stronger claim, namely, outcome-based rewards, overlap-aware off-policy evaluation, separate recording of event and available time, target-trial emulation for initiation, and silent prospective running before any interventional study, come from the wider methodological literature and remain untested in this setting. Until they are met, investigators should state which decision node a model addresses and what was knowable at that node, and readers, editors, and reviewers should not treat an improvement in simulated reward as a clinical result.
